# Seroprevalence of West Nile virus in horses in different Moroccan regions

**DOI:** 10.1002/vms3.71

**Published:** 2017-09-12

**Authors:** Abdennasser Benjelloun, Mehdi El Harrak, Paolo Calistri, Chafiqa Loutfi, Hafsa Kabbaj, Annamaria Conte, Carla Ippoliti, Maria Luisa Danzetta, Bouchra Belkadi

**Affiliations:** ^1^ Laboratory of Microbiology and Molecular Biology Faculty of Science University Mohammed Rabat V BP 1014 Morocco; ^2^ Central Command Post LGA Route de Zaer Rabat BP 5039 Morocco; ^3^ BIOPHARMA Laboratory Av. Hassan II Rabat 10100 Morocco; ^4^ Istituto Zooprofilattico dell'Abruzzo e del Molise ‘G. Caporale’ Via Campo Boario 64100 Teramo Italy

**Keywords:** West Nile, seroprevalence, horses, Morocco

## Abstract

West Nile virus‐associated disease is one of the most widespread vector‐borne diseases in the world. In Morocco, the first cases were reported in horses in 1996 and the disease re‐emerged in 2003 and in 2010. The objective of this work was to study the epidemiological situation of WNV‐associated infection in Morocco, by quantifying the seroprevalence of anti‐WNV IgM and IgG antibodies in horses in different bioclimatic regions‐zones of Morocco in 2011. During the months of May, June and July 2011, 840 serum samples were collected from horses in four regions characterized by different environmental and climatic features such as altitude, temperature and precipitation. These environmental‐climatic regions are: the Atlantic plateaus of the Gharb and pre‐Rif region, the North Atlasic plains and plateaus region, the Atlas Mountains and pre‐Atlas region and the plains and plateaus of the Oriental region. All samples were tested for the anti‐WNV IgG antibodies by ELISA and positive sera were confirmed by virus neutralization (VN). An anti‐WNV antibody prevalence map was developed. A total of 261 samples (31%) were found positive by both techniques. The prevalence of the infection was higher in the Atlantic plateaus of the Gharb and pre‐Rif region, in the northern part of the country. Available data concerning the previous WNV‐associated disease outbreaks in Morocco and the preliminary results of this serological survey suggest that the Moroccan northwest is the region at highest risk for WNV circulation. In this region, the climate is more humid with higher rainfall than other regions and milder winter temperatures exist. In the same area, the presence of migratory bird settlements may affect the risk of virus introduction and amplification.

## Introduction

West Nile Virus (WNV) is a flavivirus belonging to the *Flaviviridae* family and to the Japanese encephalitis serocomplex which contains a number of viruses causing encephalitis in humans and animals (Petersen & Roehrig 2001). WNV is common in Africa, Europe, Middle East, North America and West Asia.

In nature, WNV is maintained by cycling through birds and mosquitoes; numerous avian and mosquito species are known to support virus replication. Birds represent the vertebrate amplifying hosts (Komar *et al*. 2001), responsible for the virus maintenance in the environment and migratory species can be considered the main responsible for the geographical spread of the infection (Calistri *et al*., 2010). Humans, equines and other mammals are regarded as incidental or dead‐end hosts.

The epidemiological cycle of WNV‐associated disease is supported by avian reservoirs and vectors, mainly *Culex* mosquitoes where the virus follows a cycle of intrinsic development (Hubalek & Halouzka 1999). The temperature has a great influence on the speed of the cycle as well as on female *Culex* survival and also on the duration of the transmission period. In the avian hosts, after infection, the virus causes a viraemia lasting few days in competent avian hosts (in particular, in the order Passeriformes) (Komar *et al*. 2003).

When local ecological conditions are suitable for virus amplification, the infection in humans and horses can occur. They are considered as accidental and dead‐end hosts. Affected horses frequently demonstrate mild to severe ataxia. Signs can range from slight incoordination to recumbency. Some horses exhibit weakness, muscle fasciculation and cranial nerve deficits. Fever is not a consistently recognized feature of the disease in horses (OIE Terrestrial manual, 2013).

In Morocco, in 1996, the first outbreak was reported in 42 horses with a case‐fatality rate of 44% (Tber^1996^). After an apparent epidemiological silence, WNV‐associated disease re‐occurred in 2003 (Schuffenecker *et al*. 2005) when nine equine cases (with five deaths) were reported. In 2010, in central and north‐western part of the country, WNV‐associated disease was confirmed in 17 horses (with eight deaths) (WAHID, 2010).

The circulation of West Nile virus has been reported in wild birds in north‐east Kenitra city in 2008, in particular anti‐WNV antibodies were detected in blood samples from Blackbirds (*Turdus merula*), House Sparrows (*Passer domesticus*) and Cetti's Warbler (*Cettia cetti*) (Figuerola *et al*. 2008). Evidence of human infection was confirmed in 1996 by the death of one person due to West Nile neuro‐invasive disease (El Harrak *et al*. 1997) and in 2011 by the detection of neutralizing antibodies in 59 among 499 tested persons (11.8%) close to Meknes, Rabat and Kenitra cities (El Rhaffouli *et al*. 2012).

The WNV isolates in Morocco belong to lineage1, clade 1a, cluster 2 and more precisely to the Western Mediterranean subtype grouping isolates from France, Portugal and Italy (May *et al*. 2011). Lineage 1 isolates are found in northern and central Africa, Israel, Europe, India, Australia (Kunjin virus), North and Central America, and in Columbia and Argentina in South America. Lineage 2 strains are endemic in central and southern Africa, and Madagascar, although their presence was notified in the last years in Russia and other Eastern European countries.

Eco‐climatic variables such as rainfall and vegetation indices were shown to be associated with the occurrence of WNV‐associated disease in Morocco in 2003 and 2010 (Calistri *et al*. 2013).

The objective of this work was to study the epidemiological situation of WNV‐associated infection in Morocco, by quantifying the seroprevalence of anti‐WNV IgM and IgG antibodies in horses in different bioclimatic regions‐zones of Morocco in 2011.

## Material and Methods

### Sampling strategy, study area and sample size

A cross‐sectional study was performed during the months of May, June and July 2011 to quantify the serological prevalence of WNV‐associated disease and to investigate, if possible, recent exposure to the virus occurred. The sampling was conducted in the aftermath of the epizootic that occurred in Morocco in 2010 during various sport competitions held in different regions of Morocco. The sampling was targeted mostly in the areas holding the competitions that had been affected by epizootics in 1996, 2003 and 2010. As no precise data on the geographical distribution of the horses were available in Morocco, no sampling plan was possible to arrange. Instead, the maximum number of horses whose owners gave permission for blood samples to be taken were tested. A total of 840 samples were collected from healthy male and female horses over 3 years old, of various breeds, with no clinical signs related to the WNV‐associated disease. All the horses were identified and the owners declared they spent all their life in the respective zones where they were sampled in order to assign to each sample a specific zone they came from; all of them were only sampled once. However, horse owners were unable to give reliable information on the vaccination status against WNV.

**Figure 1:**
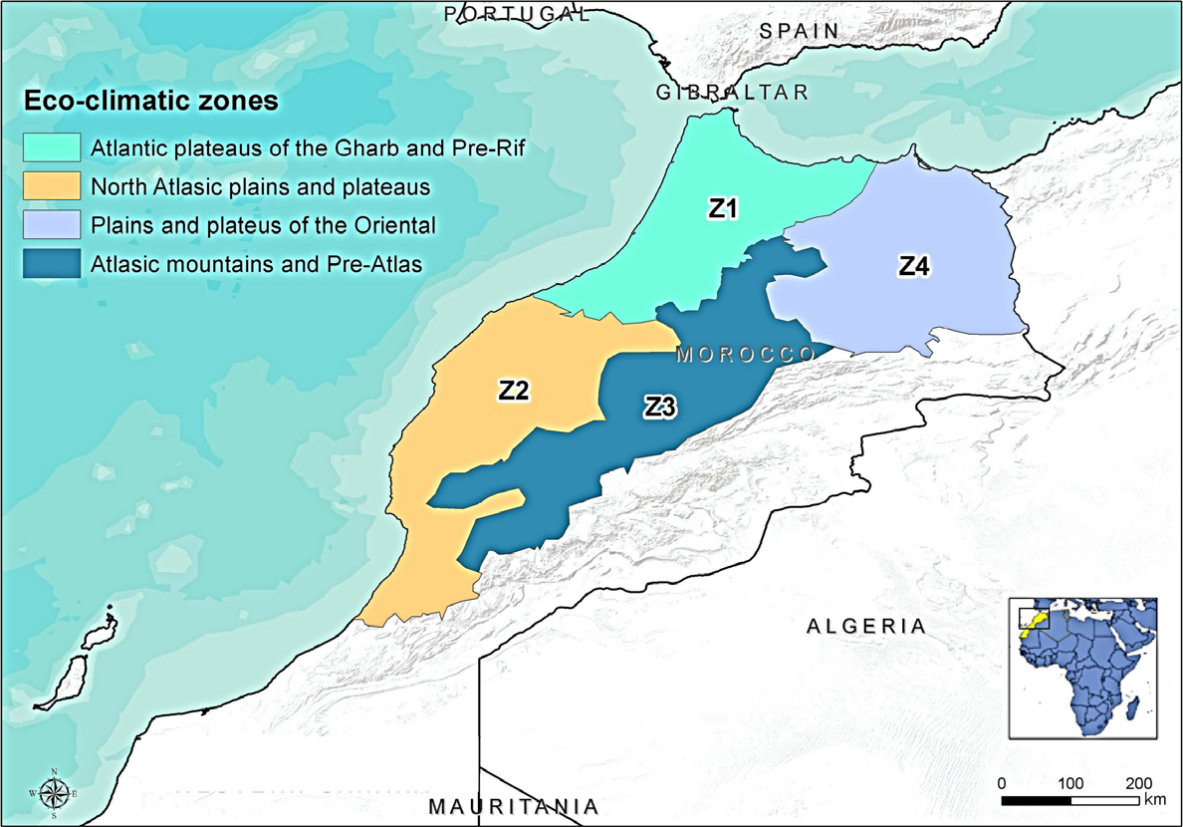
Study area with the four eco‐climatic zones concerned by the sampling.

The eco‐climatic division made by the High Commission for Water, Forests and Desertification Control (HCEFLCD, 2012) was used to assign horses to four Moroccan distinct regions‐zones, hereinafter referred to as Zone 1 ‐ Z1, Zone 2 ‐ Z2, Zone 3 ‐ Z3 and Zone 4 ‐ Z4 as reported in Fig. 1. The zones differ in terms of rainfall, altitude and temperature. The data were reported to characterize the zones. The Rif and the Gharb region were aggregated in Z1 as these zones are very similar.

The average temperature (in degrees C) and the monthly precipitation (in millimetres) were extracted as mean values for each zone Z1–Z4 from WorldClim 2 database (Fick & Hijmans 2017), through ESRI^®^ ArcGIS ArcMap 10.1 software, Spatial Analyst extension. The WorldClim 2 database aggregated across a target temporal range of 1970–2000, providing information at 1‐Km spatial resolution. Altitude values have been derived from Global 30 Arc‐Second Elevation database available from U.S. Geological Survey's EROS Data Center in Sioux Falls, South Dakota (https://lta.cr.usgs.gov/GTOPO30).

The number of sampled horses was allocated for each of the four zones according to the following scheme: from Z1 (the Atlantic plateaus of the Gharb region and pre‐Rif), 416 horses were sampled; from Z2 (the North Atlasic plains and plateaus) 196 horses were sampled, from Z3 (the Atlas mountains and pre‐Atlas region) 115 horses were sampled; and from Z4 (the plains and plateaus of the Oriental) 113 horses were sampled.

### Serological assays

For each horse sampled, blood was collected into dry tubes and transported at 4°C to Biopharma Laboratory in Rabat within 24 h, where they were centrifuged. The serum aliquots were separated and stored at −20°C until use.

As recommended by the OIE Terrestrial Manual 2013, the ELISA technique was used first to give an indication of the presence or absence of anti‐WNV antibodies in horses’ sera. Confirmation was made by the virus neutralization technique (VNT). This technique is more specific and reduces false positive results as it not only detects WNV antibodies but also gives the precise titre as the wells are scored for the observed degree of the cytopathic effect (CPE).

All samples were tested for IgG antibodies against WNV using a commercial ELISA kit (IDvet Innovative Diagnostics, reference: WNC ver. 0111 GB).

After the validation, for each sample, results were interpreted according to the manufacturer, the optical density was observed and S/N ratio (Sample optical density/Negative control optical density *100) calculated. Samples presenting a S/N ratio less than or equal to 40% were considered positive, while those with a S/N ratio less than or equal to 50% and greater than 40% were considered doubtful, and those with a S/N ratio greater than 50% were considered negative. In addition to the positive and negative controls of the manufacturer, internal control sera were also used as a tracer according to quality assurance system of the laboratory.

Positive and doubtful sera for anti‐WNV IgG antibodies were then tested by VNT using «Morocco 96.111» isolate (El Harrak *et al*. 1997) for confirmation.

This technique was performed in a volume of 50 *μ*L in cell culture micro plates, using four wells per serum dilution. After inactivation, sera were threefold diluted and mixed with an equal volume of the virus containing 100 infectious doses 50% (TCID50). Plates were then incubated at 37°C with 5% CO_2_ for 1 h. Positive and negative control sera were included in each test. Vero cells grown in Dulbecco's Modified Eagle's Medium were supplemented with 5% foetal calf serum and added to obtain confluence in 48 h.

Back‐titration of 100 TCID50 doses was performed for test validation. Reading was carried out on the fifth day by observation of the CPE and the serum titre was calculated according to Reed and Muench method (Reed & Muench 1938). Sera with a neutralizing titre equal or greater than 1:10 were considered positive.

To evaluate recent exposure to WNV, sera were tested for IgM antibodies by ELISA using IDVet Innovative Diagnostic kit (reference: WNIGM ‐ 1P ‐4P WNIGM). Because of reagent availability, only 30% of VNT‐positive sera, originating from regions where previous WNV associated disease outbreaks occurred, were tested for IgM antibodies (*n* = 78). These regions are Benslimane (18 horses), Kenitra (17 horses), Larache (17 horses), Sidi Kacem (23 horses) and Sidi Slimane (3 horses).

### Data analysis

Data collected was analysed using Microsoft Excel^®^ spreadsheet. Each sample was considered to be positive if it tested positive to ELISA IgG and then confirmed with VNT. Also doubtful samples confirmed by VNT were considered as positives for seroprevalence calculation. The seroprevalence in each of the four zones and 95% confidence intervals (CI) was calculated using a Bayesian approach based on Beta distribution *β* (*n* + 1; *n*‐s + 1) for each probability value (Table 1). Seroprevalence were also geographically represented according to the municipalities from where the samples were collected through ESRI^®^ArcMap10.1 software.

**Table 1:** Results of the serological assays (IgG ELISA and VNT) by zone

	TESTED	ELISA+	ELISA‐	DOUBTFUL	ELISA+ VNT+	ELISA+ VNT‐	Total ELISA+and DOUBTFUL	Total VNT+	Total VNT‐	Prevalence (%)
Z1	416	202	212	2	183	19	204	183	21	44.0 [39.3–48.8]
Z2	196	39	157	0	26	13	39	26	13	13.3 [9.2–18.7]
Z3	115	30	84	1	26	4	31	27	4	23.5 [16.7–32.0]
Z4	113	27	86	0	25	2	27	25	2	22.1 [15.5–30.6]
TOTAL	840	298	539	3	260	38	301	261	40	31.1 [28.0–34.3]

Furthermore, the probability distribution of the seroprevalences in the four Moroccan regions was compared using the same Bayesian statistical approach, where *n* = 840 is the total number of tested samples and *s* = 261 are those tested positive with VNT. The 95% confidence intervals were calculated for each probability value.

## Results

Out of the 840 sera tested by ELISA IgG, 298 tested positive, 539 negative and 3 were doubtful. Positive and doubtful sera (301) were tested by VNT to confirm the presence of anti‐WNV antibodies and a total of 261 horses were confirmed positive by VNT (Table 1). The one doubtful sample in Z3 was confirmed by VNT while two doubtful samples obtained from Z1 were not confirmed positive by the same technique.

The total number of samples not confirmed by VNT was 40 (of which 38 samples previously tested positive by ELISA IgG and 2 previously tested doubtful). Therefore, the percentage of sera tested ELISA positive and confirmed by VNT was 9.4% in Z1, 33.3% in Z2, 13.3% in Z3 and 7.4% in Z4 12.

Among confirmed positive sera by VNT, 219 (84%) had a neutralizing antibody titre ≥1:30. All the sera tested negative in the IgM capture ELISA test.

The results showed that the overall seroprevalence was 31.1% (95% C.I.: 28.0–34.3%). The highest seroprevalence was found in Z1 (North West Atlantic plateaus) with 44.0% (95% C.I.: 39.3–48.8%). The lowest seroprevalence, 13.3% (95% C.I.: 9.2–18.7%) was observed in Z2 (North Atlas plains and plateaus). In Z3 (Atlas mountains region), the prevalence was 23.5% (95% C.I.: 16.7–32.0%) and 22.1% (95% C.I.: 15.5–30.6%) in the plains and plateaus of the Oriental (Table 1).

**Figure 2:**
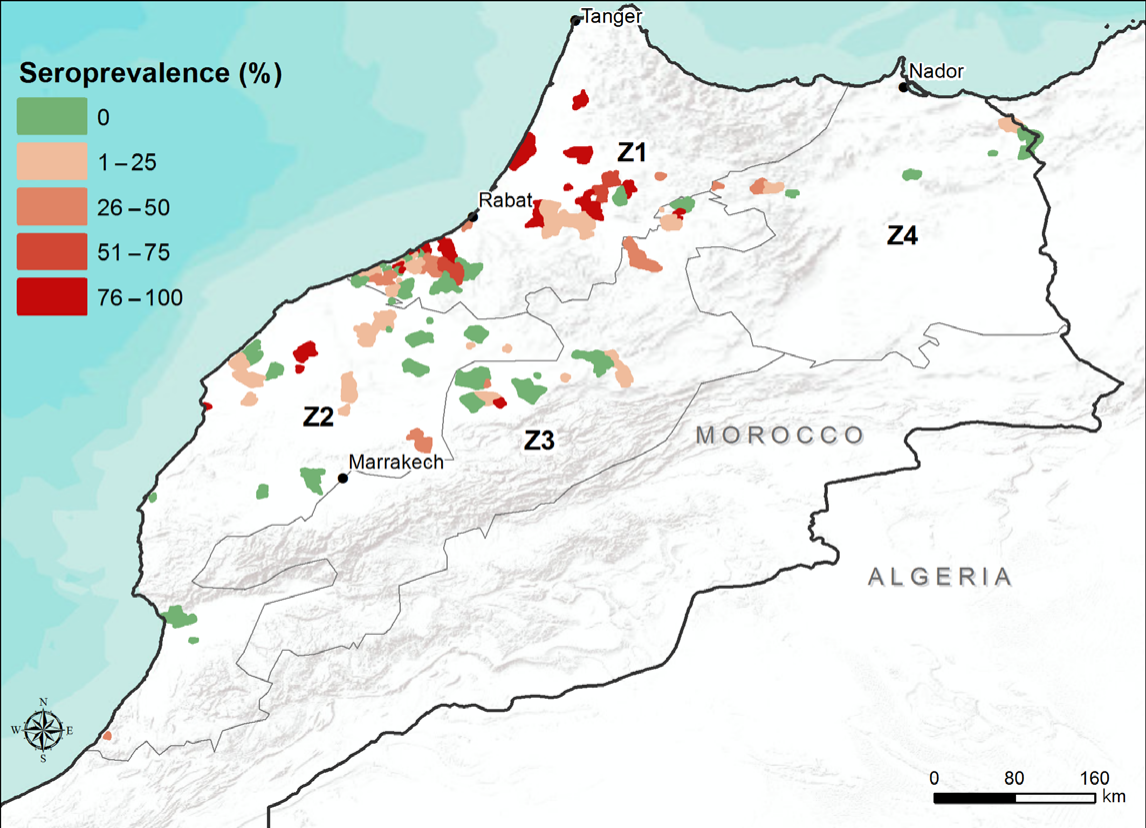
Geographical distribution of the municipalities and their prevalence.

The seroprevalence was also represented geographically by sampled municipalities in the four zones as in Fig. 2. A total of 97 municipalities were sampled, of which 44 were in Z1, 29 in Z2, 16 in Z3 and 8 in Z4.

**Figure 3:**
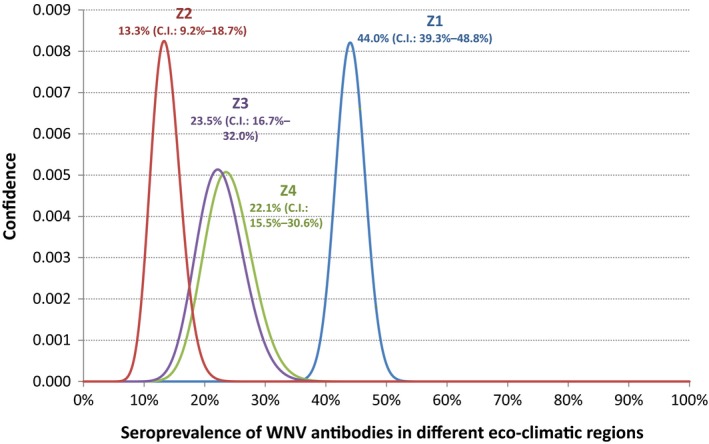
Probability distribution of the seroprevalence in the four zones.

According to a Bayesian approach, the seroprevalence level in Z1 was statistically different from the others regions. The three zones Z2, Z3 and Z4 showed lower seroprevalence with no significant differences among them, as their 95% confidence interval overlap each other (Fig. 3).

## Discussion

According to the results of the serosurvey, 31.1% of tested horses were positive to anti‐WNV IgG antibodies. Out of 261 sera, 219 (84%) had a neutralizing titre higher than 1:30.

The difference between the results obtained by ELISA and VNT techniques can be attributed to the high sensitivity of ELISA test (the proportion of infected horses that tested positive for WNV IgG antibodies) compared to the high VNT specificity (the proportion of non‐infected horses that tested negative).

Such difference could also be due to the presence of WNV cross‐reactive sera; close antigenic relationships between WNV and other members of the Japanese encephalitis group is indeed responsible for cross‐reactions in rapid serological tests such as ELISA (Hirota *et al*. 2010). Concomitant circulation of WNV and other flaviviruses can make the result interpretation difficult. The difficulty of interpretation attributed to cross‐reactions among viruses from JE serocomplex has been encountered in India and Pakistan where WNV and Japanese encephalitis virus co‐exist in humans with similar symptoms, and in North America where WNV co‐exists with St. Louis encephalitis virus (Zientara *et al*. 2009). In Morocco, circulation of other flaviviruses should also be taken in consideration as antibodies against Usutu virus were found in wild birds in Kenitra region in Z1 (Figuerola *et al*. 2008). The circulation of other flaviviruses may explain the overall observed 12.7% of ELISA test positive but negative VNT samples. Consequently, it would be relevant for further studies to investigate in depth the likelihood of cross‐reactivity with other flaviviruses in Morocco, including all the viruses belonging to the Japanese Encephalitis serocomplex, as for example, by means of control samples, that is, true positive sera from horses known to be infected with Usutu virus.

The observed titre of the positive sera is probably due to the re‐infection effect during the previous 2010 outbreak or a prolonged and repeated exposure to circulating viruses in endemic areas. The absence of IgM‐positive serum, that could demonstrate a recent infection during the last 3 months, may be explained by the fact that the samples were taken outside the endemic season.

During the 2010 outbreak, a limited number of vaccine doses were used in some areas. One vaccine contained a lyophilized recombinant canarypox‐vectored WNV (Recombitek^®^ Equine WNV, MERIAL) and another inactivated vaccine (WNVac Biopharma).

A possible confounding effect of vaccination against WNV in the equine population on the seroprevalence results reported may represent a limitation to this study. Therefore, in the absence of a precise official data on vaccination against WNV, we calculated the ratio of the number of vaccine doses distributed by BIOPHARMA National laboratory, as controlled by Health authorities (data not published) to the number of horses counted by the Ministry of Agriculture (Benazzou & Benlekhal 2005). As a result, the overall immunization rate is approximately 8%. This rate may vary in the epizootic areas of 2010 where limited vaccination had been carried out around the outbreaks as notified to the OIE and may influence the prevalence obtained. Conversely, a serological survey, conducted in the absence of any vaccination after the outbreaks occurred in 2003 in Z1, showed a global prevalence of 45% in different equid species (Iraqui 2006) which supports our results and minimizes this limitation.

Another limitation is the confounding effect of animal movements between the four studied zones. Horses’ displacements in Morocco are generally limited to attending traditional sport competitions for short periods of time. Therefore, any impact on the anti‐WNV antibody prevalence should be minimal.

Our data suggest that the horses from Atlantic plateaus of the Gharb and pre‐Rif (Z1) have the highest prevalence of WNV infection, while the seroprevalence was significantly lower in the other three regions (the Atlas Mountains and pre‐atlas region, the plains and plateaus of the Oriental and the North Atlas plains and plateaus). This finding is consistent with the previous reported WNV‐associated disease outbreaks in Morocco. Indeed, the north‐western Atlantic region was involved in the majority of notified cases in horses during 1996 (Tber 1996), Tber *et al*. 2003; (Schuffenecker *et al*. 2005) and 2010 outbreaks (WAHID, 2010).

WNV‐associated disease epidemics are clearly linked to the abundance of vectors and the availability of susceptible bird populations considered as the virus reservoir (Conte *et al*. 2015).

In Morocco, 42 species of mosquitoes have been listed; they belong to Anophelinae and Culicinae families including members of *Culex pipiens* complex (Trari *et al*. 2003). The *Culex pipiens* complex includes *Cx. pipiens* and *Cx. quinquefasciatus*. *Cx pipiens* is the most widely distributed mosquito species in the Maghreb (Rioux, 1958; Krida *et al*. 1997) and its competence in transmitting WNV have been proved (Faraj *et al*. 2006; Amraoui *et al*. 2012).

Concerning bird populations, Morocco is certainly one of the richest countries in birds of the Western Palaearctic (Europe, North Africa and the Middle East). Morocco's avifauna comprises 309 migratory species, including 80 water bird species. They belong to the families of Podicipedidae, Phalacrocoracidae, Ardeidae, Phoenicopteridae, Anatidae, Accipitridae, Falconidae, Rallidae, Recurvirostridae, Charadriidae, Scolopacidae, Laridae, Sternidae, Alaudidae, Motacillidae, Turdidae, Sylviidae, Corvidae, Sturnidae and Emberizidae. (Dakki *et al*. 2011).

Eco‐climatic variables such as rainfall and vegetation indices were shown to be associated with the occurrence of WNV‐associated disease in Morocco in areas where the disease has been observed. Thus, Normalized Difference Vegetation Index (NDVI) values recorded in 2003 and 2010, from June to November, were significantly higher than those registered over the same period in non‐epizootic years of the decade 2001–2010 (Calistri *et al*. 2013). Furthermore, rainfall data showed higher peaks in 2003 and 2010, when the number of days with extreme rainfall was significantly higher than the rest of the same decade during 1–2 months before the occurrence of WNV‐associated disease cases (Calistri *et al*. 2013).

**Figure 4:**
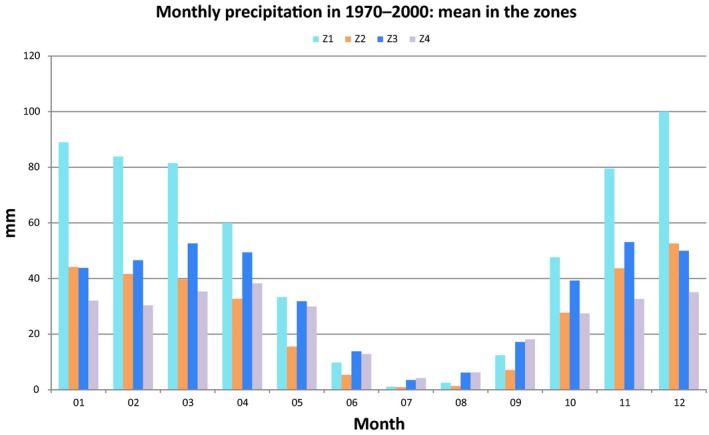
Monthly precipitation: average value reported in the zones in the period 1970–2000.

**Figure 5:**
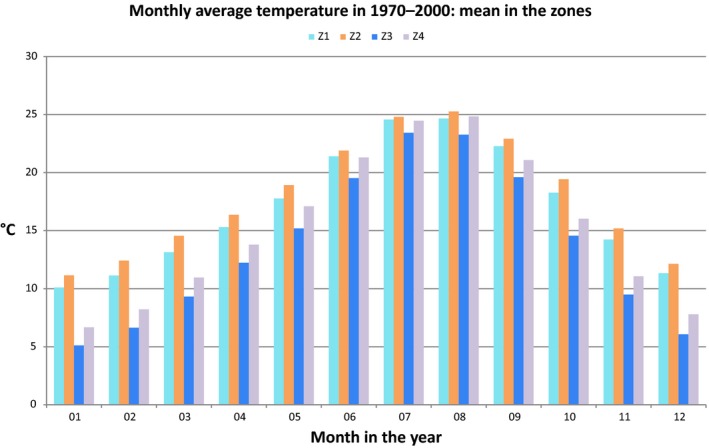
Monthly average temperature: mean value reported in the zones in the period 1970–2000.

The Atlantic plateaus of the Gharb and pre‐Rif (Z1) have a humid climate, a high rainfall level that may reach 850 mm/year (Driouech 2010), and a mild winter and summer temperatures (Figs. 4, 5). This lowland area (mean altitude is 414.3 m a.s.l., 95% C.I. 411.4–417.2), includes several wetlands (*Ramsar Convention of Wetlands,* 2005), rice fields and two natural bird reserves along a major migratory Europe sub‐Saharan flyway. An increase in the diversity of birds brings together hosts with a complementary role in the circulation of the virus (e.g. reservoir or introducer individuals and other very good amplifiers) that promotes amplification (Dobson and Foufopoulos 2001).

These bioclimatic conditions are optimal for the survival of the vector and the circulation of WNV (Calistri *et al*., 2010). In this zone, a high density of *Culex pipiens* exists besides other mosquito species such as *Anopheles labranchiae* (Marc *et al*. 2016).

The North Atlas plains and plateaus (Z2) face the Atlantic Ocean in the southwest of Morocco and the region has a mean altitude of 479.9 m a.s.l. (95% C.I.: 477.6–482.3); the climate is semi‐arid to arid and the precipitation very scarce (Figs. 4, 5). Such environmental features are less favourable for WNV cycle (Calistri *et al*. 2013).

In central Morocco, the Atlas Mountains and pre‐Atlas (Z3) has a mean altitude of 1494.7 m a.s.l. (95% C.I. 1490.2–1499.1) (https://lta.cr.usgs.gov/GTOPO30) with large temperature differences between seasons (Fig. 5).

The plains and plateaus of the Oriental (Z4) region faces the Mediterranean Sea in the north of the Country: the mean altitude is 1013.4 m a.s.l. (95% C.I. 1010.3–1016.5) and rainfall is very scarce (Fig. 4), winters are mild but summers are dry and hot (Fig. 5).

## Conclusion

Available data concerning the previous WNV‐associated disease outbreaks in Morocco and the preliminary results of this serological survey suggest that the Moroccan northwest is the region at highest risk for WNV circulation. The WNV antibody prevalence is indeed higher in the Atlantic plateaus of the Gharb and pre‐Rif region coast where conditions are suitable for the introduction, the spread and the maintenance of WNV. Therefore, a permanent surveillance system on mosquito vectors, bird populations and horses should be implemented firstly in this area to better understand WND epidemiology. Since the infection by WNV was also described in humans in Morocco (Benjelloun *et al*. 2016) in the Gharb region and in the areas of Rabat, Kenitra and Meknes, a defined surveillance systems embracing different components and cooperative integration of both human and animal health should be considered by Veterinary and Public Health Authorities at national level.

## Sources and Manufacturer

IDvet innovative diagnostics Kit, reference: WNC ver. 0111 GB. IDVet innovative diagnostic kit (reference: WNIGM ‐ 1P ‐4P WNIGM).

## Source of Funding

This study was supported by the University Mohammed V, Science Faculty Rabat Morocco.

## Conflict of Interest

The authors declare that they have no competing interests.

## Ethics Statement

The authors declare that all institutional and national guidelines for use of horses sampled in this study were followed.
